# Implementation of data protection laws in the European Union and in California is associated with a move of clinical trials to countries with fewer data protections

**DOI:** 10.3389/fmed.2022.1051025

**Published:** 2022-11-10

**Authors:** Elad Yom-Tov, Yishai Ofran

**Affiliations:** ^1^Microsoft Research Israel, Hertzliya, Israel; ^2^Faculty of Industrial Engineering and Management, Haifa, Israel; ^3^Hematology and Bone Marrow Transplantation Department, Sharee Zedek Medical Center, Jerusalem, Israel; ^4^Faculty of Medicine Hebrew University of Jerusalem, Jerusalem, Israel

**Keywords:** GDPR - General Data Protection Regulation, CCPA, Consumer Privacy Act, clinical trials, data protection and privacy

## Abstract

The European Union implemented data privacy laws in mid-2018 and the state of California enacted a similar law several weeks later. These regulations affect medical data collection and analysis. It is unclear if they achieve this goal in the realm of clinical trials. Here we investigate the effect of these laws on clinical trials through analysis of clinical trials recorded on the US's ClinicalTrials.gov, the World Health Organization's International Clinical Trials Registry Platform and scientific papers describing clinical trials. Our findings show that the number of phase 1 and 2 trials in countries not adhering to these data privacy laws rose significantly after implementation of these laws. The largest rise occurred in countries which are less free, as indicated by the negative correlation (−0.48, *p* = 0.008) between the civil liberties freedom score of countries and the increase in the number of trials. This trend was not observed in countries adhering to data privacy laws nor in the paper publication record. The rise was larger (and statistically significant) among industry funded trials and interventional trials. Thus, the implementation of data privacy laws is associated a change in the location of clinical trials, which are currently executed more often in countries where people have fewer protections for their data.

## Introduction

The European Union's General Data Protection Regulation (GDPR) is a regulation on data protection and privacy implemented in May 2018 (1). In September 2018, California passed the Consumer Privacy Act (CCPA) (2), which similarly regulate protections for people's data. The law went into effect on January 2020. These regulations attempt to provide people with stronger protections for their data and to govern how medical data, among other categories, is collected, stored, and analyzed.

GDPR has special provisions which aim to enable scientific research (Article 89), but even under these provisions, significant requirements are placed on researchers (3). Moreover, these regulations suffer from lack of regulatory clarity and inconsistent implementation (4, 5). Thus, in the past few years, several researchers have raised concerns about limitations on research imposed by GDPR, especially with regard to archival (secondary) use data (6), cross-border trials (7), and pediatric trials (8).

Clinical trials differ in the medical databases and personal health-related information that they use. Some trials rely on data collected by electronic systems as part of the regular operation of the medical system. These data are collected at the location where patients are treated and adhere to the storage protocols of health providers or insurer organizations. Other trials collect their own data as part of prospective clinical trials of all phases. Their creation requires a laborious and expensive effort of meticulous follow-up and recording. While both types of trials are affected by data protection regulations, we hypothesize that it is easier to move trials which collect their own data to other locations, since their data does not yet exist, and they do not rely heavily on existing electronic patient records.

In a global research community, sponsors and researchers from countries implementing data protection regulations may have the ability to move their trials to countries where such regulations do not exist. Thus, here we attempt to test if such a trend can be identified from the data. To do so, we analyzed records of clinical trials before and after the implementation of GDPR and CCPA to evaluate which of these courses the research community at large has taken. We compared those results to the scientific papers published over the same period to test if similar effects are evident in the publication record.

## Methods

We analyzed all trials registered on ClinicalTrials.gov until July 31, 2021. Even though this repository is maintained by the US government it records trials from many countries around the world and is the repository recommended by the International Committee of Medical Journal Editors (9). As a comparison, we analyzed all trials recorded on the World Health Organization's International Clinical Trials Registry Platform (ICTRP, https://www.who.int/clinical-trials-registry-platform, accessed June 12th 2022). For the latter, the date of a trial was recorded as the enrollment date and, if that date was not provided, the registration date.

We defined the data protection implementation (DPI) date as May 25, 2018. This is the implementation date of GDPR, but as CCPA was enacted at a similar date (June 28, 2018), we chose the earlier date for all analyses. Note that since CCPA went into effect in January 2020 we expect the effect of CCPA to be delayed compared to that of GDPR.

Countries that implement GDPR include European Union members and European Economic Area members (10). Several other countries (Andorra, Argentina, Canada, the Faroe Islands, Guernsey, Israel, the Isle of Man, Jersey, New Zealand, Switzerland, Uruguay, Japan, the United Kingdom, and South Korea) (11) were deemed by the the EU to offer adequate levels of data protections. In the following analysis these are grouped with the GDPR-implementing countries even though they do not explicitly implement GDPR, since they may place similar limitations on data to those in GDPR-implementing countries. Separate analysis for the two groups of countries is provided in the Appendix.

We assumed that trials in the US are subject to CCPA, as many organizations have implemented CCPA for all US customers, not just those in California (12, 13). Analysis of trials according to the US states in which they occurred is provided in the Appendix.

Trials related to COVID-19 were identified as those containing the words “COVID-19” or “SARS-COV-19” in the keywords of the ClinicalTrials data or the scientific title of the ICTRP. The phase was identified from the phase field in the ClinicalTrials and ICTRP repositories.

Additionally, we analyzed all papers which appear in the PubMed repository (https://pubmed.ncbi.nlm.nih.gov/). Papers related to COVID-19 were identified as those containing the words “COVID-19” or “SARS-COV-19” in the title or abstract. Similarly, papers pertaining to a trial phase were identified if a trial phase was mentioned in the title or abstract through the appearance of the phrase “phase X,” where X is 1, 2, or 3 (in both Arab and Roman numerals). If more than one trial phase name was identified, the paper was excluded from our analysis. The country affiliation of the first author and the most common affiliation of the authors were also captured. Appendix 5 reports a similar analysis, focusing solely on articles identified by PubMed as reporting clinical trials.

The freedom scores of countries (specifically, civil liberties scores) were taken from Freedom House's Global Freedom status, 2021 (14). At the time of writing we are unaware of an agreed-upon measure for countries' data protection. However, as protection of personal data is commonly associated with civil liberties (15) and individual rights (16), we used the civil liberties score from Freedom House as a proxy for data protection. See also the Appendix for additional measures provided therein.

We used two methods to identify if the number of trials per month changed around the date of DPI in a statistically significant manner: First, we used segmented regression (17). Specifically, we computed the difference between the actual number of clinical trials after DPI and the number predicted by a linear model trained on the number of clinical trials prior to DPI. We modeled this difference using a linear model and used the F-test to test if the coefficients of the model were different from zero. We refer to this test as a residual test. Second, we applied the Chow test for structural stability (18) to the number of trials per month.

We quantified the change in the number of clinical trials per country following DPI by calculating the percentage of trials after DPI in a country among all trials in that country. This change was correlated with country indicators such as the freedom score to assess which of these variables were associated with the change.

The statistical significance of changes in the number of trials, stratified by trial attributes, was assessed using the chi^2^ test. Bonferroni correction was made in all cases of multiple testing.

## Results

**Figure 1:**
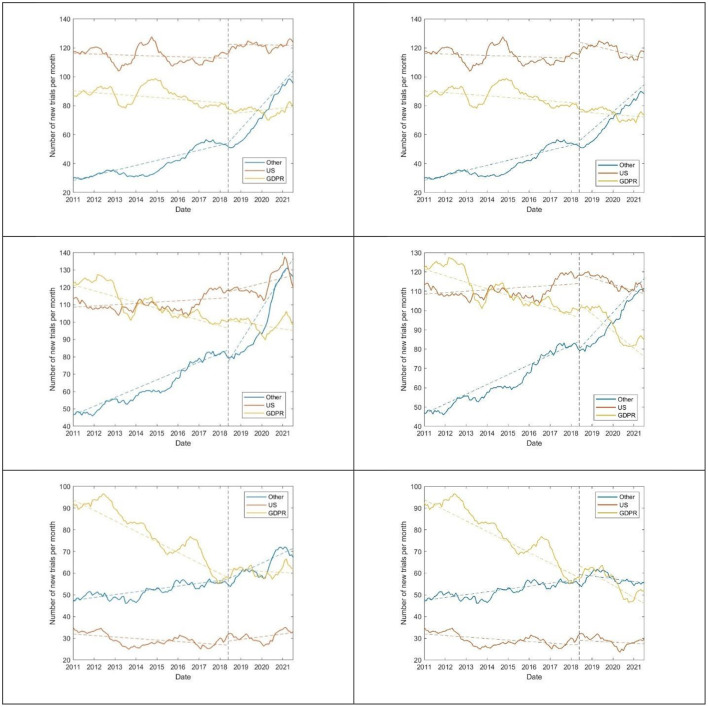
Number of new phase 1 **(top)**, 2 **(center)**, and 3 **(bottom)** clinical trials registered on ClinicalTrials.gov per month by DPI status. Graphs on the left comprise all trials, while those on the right exclude COVID-19 trials. The vertical dotted line denotes the date of DPI. Colored dotted lines are linear regression lines fit to the data.

A total of 249,387 trials were logged on ClinicalTrials.gov between January 1, 2011, and July 31, 2021. Figure 1 shows the number of new clinical trials registered per month by study phase. The number of trials in non-data protection (DP) countries (those that did not implement privacy regulations) rose more significantly after DPI (*P* < 10^−4^ for both residual and Chow tests) for phase 1 and 2 trials, but not for phase 3. The change was not statistically significant for GDPR countries and the US.

COVID-19-related trials were most commonly phase 2 trials (49%, compared to 21% phase 1 and 30% phase 3). Among all trials the number of trials in non-DP countries rose more significantly after the data protection implementation (DPI) (*P* < 10^−3^) for phase 1 and 2 trials, but not for phase 3. The change was not statistically significant for GDPR countries and the US.

**Figure 2:**
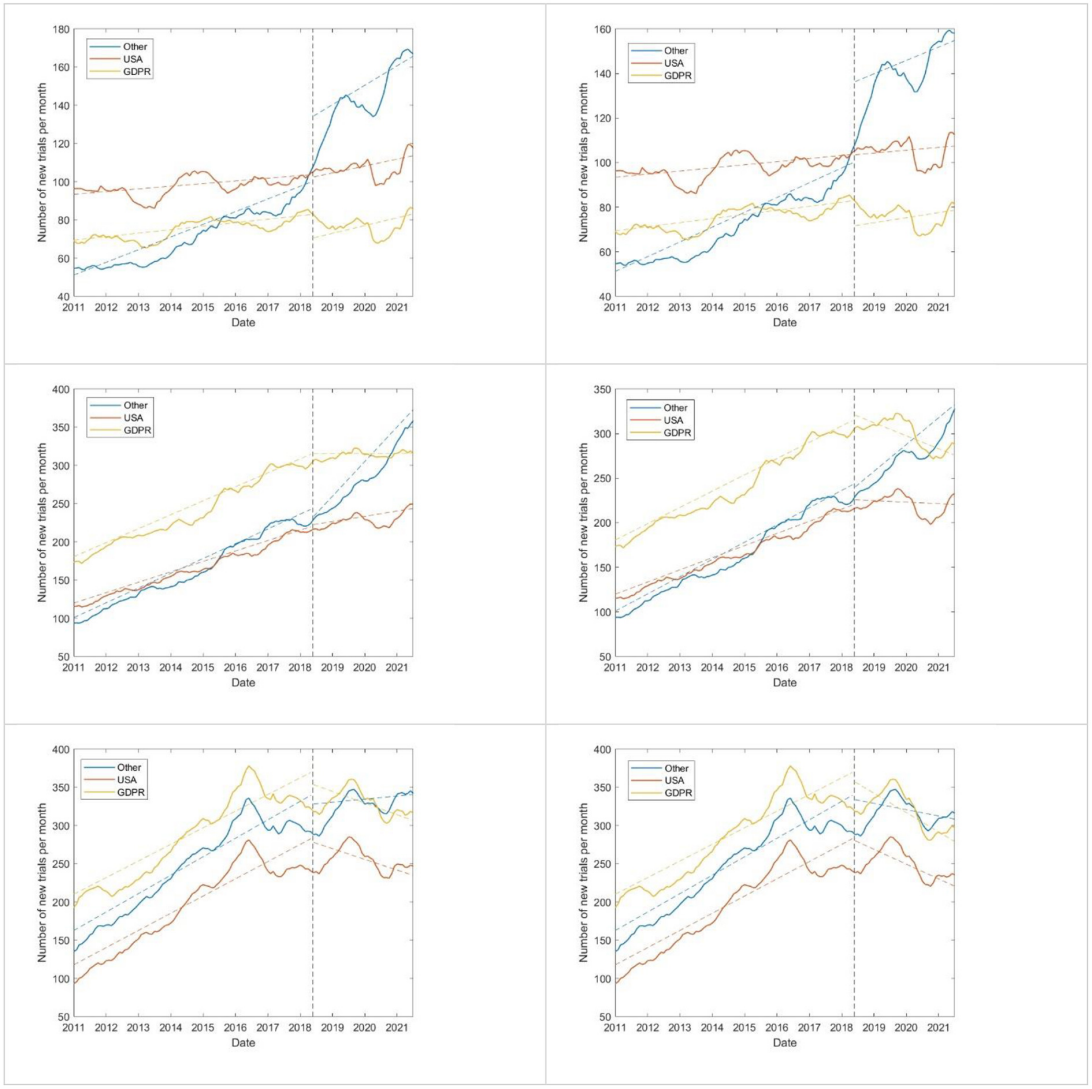
Number of new phase 1 **(top)**, 2 **(center)**, and 3 **(bottom)** clinical trials registered on ICTRP per month by DPI status. Graphs on the left comprise all trials, while those on the right exclude COVID-19 trials. The vertical dotted line denotes the date of DPI. Colored dotted lines are linear regression lines fit to the data.

The World Health Organization's International Clinical Trials Registry Platform (ICTRP) logged 578,910 trials between January 1, 2011, and July 31, 2021. Figure 2 shows the number of new clinical trials registered per month by study phase. The number of trials in non-DP countries rose more significantly after DPI (*P* < 10^−3^ for both residual and Chow tests) for phase 1 and 2 trials. The change was not statistically significant for GDPR countries and the US for phase 1, but for phase 2 in GDPR countries and phase 3 in all countries there was a statistically significant (*P* < 0.003) drop in the number of new trials. The Chow test showed a statistically significant change around DPI date for both GDPR countries and US in phase 2 trials in the US and phase 3 trials in both US and GDPR countries (*P* < 10^−3^).

Thus, data from ClinicalTrials.gov is consistent with that of ICTRP in that non-DP countries experienced a large rise in the number of trials after DPI, especially in phase 1 trials, whereas DP countries had no change or a drop in the number of their trials. As the changes in the number of trials over time among the two repositories is qualitatively similar, we henceforth focus on analysis of data from ClinicalTrials.gov.

**Figure 3:**
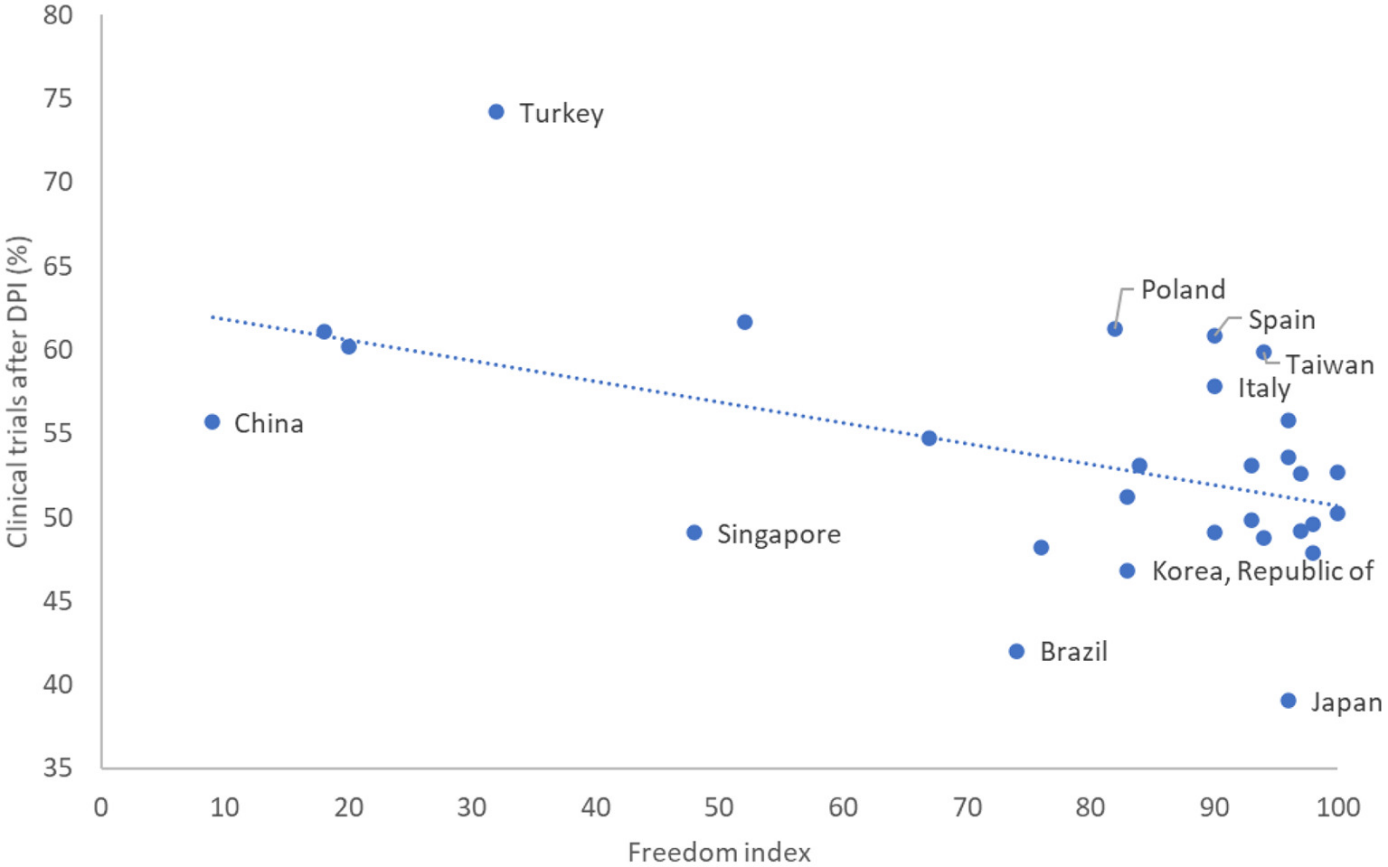
The relationship between the percentage of trials conducted after DPI (out of all trials conducted in a country) as a function of the civil liberties freedom score. The ten countries farthest from the regression line are marked.

Comparing the 2 years before DPI to the 2 years after, the five countries with at least 250 clinical trials that had the largest increase in the number of trials after DPI were (in descending order) Pakistan, Turkey, Mexico, Hong Kong, and Egypt (Turkey, Hong Kong, Egypt, Russia, and Taiwan at a threshold of 500 trials). The largest decrease in countries with DP occurred in Japan, South Korea, Finland, the Netherlands, and Israel (Japan, South Korea, the Netherlands, Israel, and Germany at a threshold of 500 trials). Figure 3 shows the relationship between the percentage of trials conducted in a country (out of all trials conducted in that country) after DPI as a function of the civil liberties freedom index. As the figure shows, there is a negative correlation between the freedom score of countries and the increase in the percentage of trials (−0.48, *p* = 0.008) following DPI, indicating that the largest rise in the number of clinical trials occurred in countries which are less free. The Appendix evaluates other measures of individual freedom, the rule of law and economic freedom for their correlation with the change in the number of trials per country.

Comparing the 2 years before and after DPI, the earlier the phase of a trial, the more likely it was to exhibit a larger decrease in percentage of trials taking place in DP countries compared to non-DP countries following DPI (0.95, *p* = 0.05).

Industry-funded trials increased 18% after DPI in non-DP countries but did not change in number in DP countries (chi-square test, *P* < 10^−10^). Other funders (e.g., NIH, US federal) showed no similar statistically significant difference.

Both randomized and non-randomized studies increased 27% (chi-square test, *P* < 10^−10^) in non-DP countries, while in DP countries, randomized studies increased 4% and non-randomized studies decreased by 4% (chi^2^ test for both, *P* < 10^−10^).

Observational studies were less likely to occur in DP countries after DPI compared to non-DP countries (14% increase in DP countries compared to 48% increase in non-DP countries). Interventional studies had a larger relative change: 3% increase in DP countries compared to 28% increase in non-DP countries (chi^2^ test, *P* < 10^−10^).

The intervention types that had the largest decrease in appearance in DP countries following DPI were behavioral, drug, and procedure. In contrast, dietary supplements, diagnostic tests, and biological products had the smallest decrease in appearance in DP countries (chi^2^ test, *P* < 0.05).

Trials which were conducted in both DP and non-DP countries increased by 2% compared to a 13% rise in those which were conducted entirely within or outside DP countries (chi^2^ test, *P* = 0.001).

**Figure 4:**
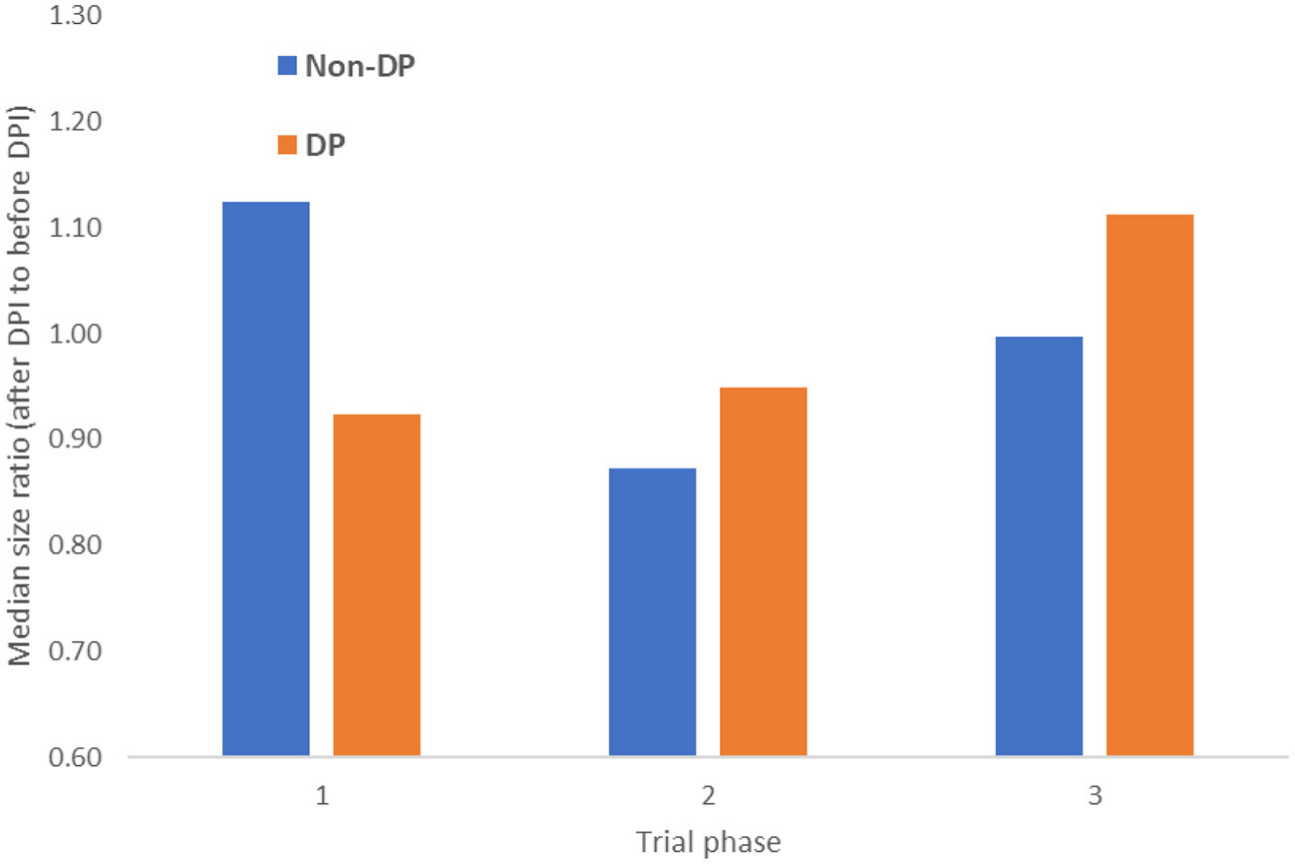
Change in the number of trial participants after DPI date by trial phase and DP status.

A comparison of the average number of participants in trials after DPI compared to before it, for DP and non-DP countries, stratified by trial phase is shown in Figure 4. As the figure shows, the number of participants in phase 1 trials grew by ~13% after DPI in non-DP countries (and were reduced by 8% in DP countries), while phase 3 trials grew by 11% in DP countries.

A total of 11,078,216 papers were published on PubMed between January 1, 2011, and July 31, 2021. Of those, the trial phase was mentioned in 47,299 papers. The country affiliation of the first author and the most common affiliation were identical in 98% of papers. Therefore, we focus on the affiliation of the first author. One hundred random papers which were identified as mentioning a phase number were inspected by one of the authors to verify that the correct phase number was identified and that the paper refers to a single phase trial. Of the sampled papers, 92% were correctly identified.

**Figure 5:**
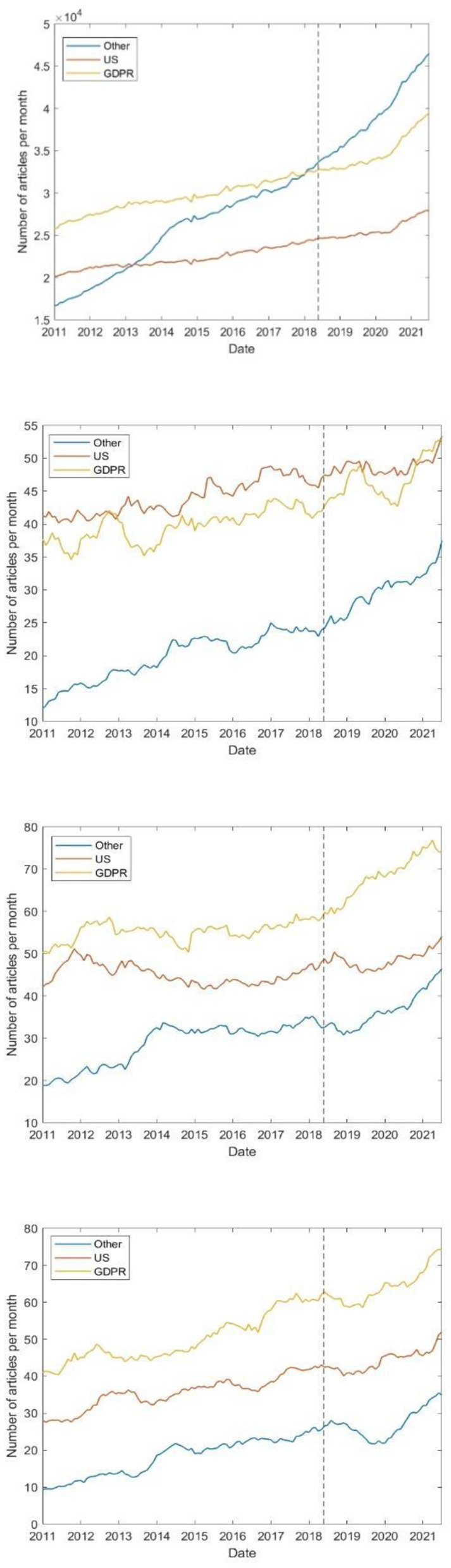
Number of papers published over time, excluding those related to COVID-19. From top to bottom: Total, phase 1, 2, and 3. The vertical dotted line denotes the date of DPI.

Figure 5 shows the number of non-COVID-19-related papers published over time, both in total and stratified by trial phase. As the figure shows, there is no statistically significant change (*P* > 0.05) in the trend of published papers following DPI.

## Discussion

The European Union and the US state of California have attempted to regulate the protection of individual's data, thus providing more protections and choice to citizens. GDPR and CCPA have been widely adopted, potentially having a significant effect on clinical trials throughout the world. Our analysis of clinical trial registrations from two sources reveals large changes in the location, size, and type of clinical trials after the date of implementation of data protection laws.

The earlier the phase of a trial, the more likely it was to shift into non-DP countries after the DPI date. The number of phase 1 and phase 2 trials in non-DP countries rose more significantly after the DPI date. No similar rise occurred for phase 3 trials or in DP countries. Countries which are ranked less free (in terms of civil liberties) experienced the largest rise in the number of clinical trials after the DPI date. The change in location was led by industry-funded trials, which increased significantly after the DPI date in non-DP countries, but did not change in DP countries.

Moreover, within GDPR countries, the number of new trials after DPI dropped in countries deemed as having “adequate protections” and flattened in GDPR countries (see Appendix 2). A similar observation is seen when comparing trials in California, in California and other US states and solely in other US states (see Appendix 3).

Phase 3 studies are large-scale operations that take a long time to conduct and launch; therefore, it may still be early to see the an association between DPI and trial locations of phase 3 trials. Moreover, phase 3 studies require the infrastructure of multiple high-volume/high-quality centers. Long-term survival results of such studies are affected by the quality of supportive care in the community, quality of treatment for comorbidities, and logistics issues; therefore, it is a challenge to conduct phase 3 studies solely in non-DP countries. The challenges for phase 1 and 2 studies are the complexity and innovation of the technique in use. Surprisingly, we did not observe a parallel shift in the nationality of the first authors of papers following DPI; thus, the fact that industry-led studies shifted to non-DP countries probably does not reflect a scientific renaissance in these countries.

Trials relying on large amounts of data, such as observational studies, were less likely to occur in DP countries after the DPI date compared to non-DP countries. Similarly, intervention types that require significant data collection, including behavioral, drug, and procedure, had the largest increase in non-DP countries.Phase 1 trials in non-DP countries grew in the number of participants, whereas the size of phase 2 and 3 trials grew in DP countries.

Trials which were conducted in both DP and non-DP countries increased less than those which were conducted entirely within or outside DP countries. We attribute this to the difficulties highlighted by several researchers as to the difficulties in data sharing following the implementation of data privacy laws [see, for example, Eiss (19), Ursin (20), and Slokenberga (21)].

These trends in location, size, and type of trials were not reflected in the paper publication record, where there is no discernible change in the trend of published papers following the date of DPI (see also Appendix 5). We attribute the change in location of trials, which is not reflected in the publication record, to what has been described as “helicopter research,” where researchers work in low income countries with little involvement of local researchers (22). While sponsors of clinical trials have influence on study locations decisions, there is no transparency or public access to the considerations that drive these decisions.

We note that, while we analyzed the two largest datasets of clinical trials, they are not a perfect record of all clinical trials. For example, researchers have noted biases in the trials recorded on ClinicalTrials.gov, including changing incentives for recording trials and the fact that recording trials is voluntary (23). For this reason, we analyzed two datasets, in the hope that this reduces the effects of these biases.

The publication record, as reflected in PubMed data, is imperfect for several reasons. First, it may be that a publication appears several years after the registration of the relevant clinical trial, and thus our data does not cover a sufficient time period after the date of DPI to show possible changes in the publication record. Second, some trials may be described by several publications released over time, which could skew the results. However, if it is assumed that the number of papers per trial remains similar before and after DPI, this should only have a small effect on the results. Finally, our identification technique could be missing some trials, for example, those not identified by phase number or referring to more than one phase in the same paper. For this reason, Appendix 5 reports the analysis of a subset of papers, identified by Pubmed as referring to clinical trials.

Taken together, our results suggest that, in parallel to the implementation of GDPR and CCPA, clinical trials, especially early ones and those requiring significant data collection, are more often conducted in countries where people have fewer protections for their data. However, the investigators remained in their original countries.

We hypothesize that the changes we observed are related to DPI, though we cannot directly prove this causal effect. However, if our hypothesis is correct, our data may offer empirical evidence for the existence of the phenomena of “ethics dumping” (22, 24), which occurs when researchers export unethical or unpalatable experiments and studies to lower-income or less-privileged settings with different ethical standards or less oversight. Interestingly, we have found that trials moved not only to lower-income countries but also to wealthy ones such as Hong Kong. This may suggest that the definition of ethics dumping should be widened to include countries where protections to individuals, whether to their privacy or to other aspects of their self, are not sufficiently rigorous.

## Data availability statement

Publicly available datasets were analyzed in this study. This data can be found here: ClinicalTrials.gov https://www.who.int/clinical-trials-registry-platform; https://pubmed.ncbi.nlm.nih.gov.

## Author contributions

Conceptualization, methodology, visualization, and writing: EY-T and YO. Analysis: EY-T. All authors contributed to the article and approved the submitted version.

## Conflict of interest

The authors declare that the research was conducted in the absence of any commercial or financial relationships that could be construed as a potential conflict of interest.

## Publisher's note

All claims expressed in this article are solely those of the authors and do not necessarily represent those of their affiliated organizations, or those of the publisher, the editors and the reviewers. Any product that may be evaluated in this article, or claim that may be made by its manufacturer, is not guaranteed or endorsed by the publisher.
